# Clinical application of the pedicle in vitro restorer in percutaneous kyphoplasty

**DOI:** 10.1186/s13018-018-0978-8

**Published:** 2018-10-25

**Authors:** Yimin Qi, Yiwen Zeng, Dalin Wang, Jisheng Sui, Qiang Wang

**Affiliations:** 1 Nanjing Medical University, Nanjing, China; 2 Department of Orthopaedic Surgery, Nanjing First Hospital, Nanjing Medical University, #68 Changle Rd, Qinhuai District, Nanjing, 210000 Jiangsu China

**Keywords:** Percutaneous kyphoplasty, Osteoporotic vertebral compression fractures, Restorer, Distraction, Reduction, Reconstruction

## Abstract

**Background:**

Percutaneous kyphoplasty (PKP) is widely applied for the treatment of osteoporotic vertebral compression fractures (OVCFs) and has achieved satisfactory clinical results. With the accumulation of clinical cases and prolonged follow-up times, the inability to reconstruct vertebral height defects has attracted more and more attention. A comparison of clinical effects was retrospectively reviewed in 72 patients who underwent simple PKP or pedicle in vitro restorer (PIVR) combined with PKP to discuss the clinical application of self-developed PIVR used in PKP.

**Methods:**

From August 2013 to August 2016, 72 patients with OVCFs were treated surgically, with 30 patients undergoing PKP (group A) and 42 undergoing PIVR combined with PKP (group B). Operation-related situations, radiological data, and related scores were compared between the two groups by corresponding statistical methods.

**Results:**

Bone cement was successfully injected into 72 vertebral bodies. Sixty-three cases were followed up for an average of 14 months. There were significant differences between the two groups in the improvement of the height of the vertebral body, sagittal Cobb angle, and visual analogue scale (VAS) 1 week after the operation (*P* < 0.05), and the improvements of group B were better than those in group A. The cement leakage ratio was significantly different between the two groups (*P* < 0.05). The Oswestry Disability Index (ODI) at last follow-up was significantly different between the two groups (*P* < 0.05). There was no significant difference in the incidence of recurrent vertebral fractures between the two groups at the last follow-up (*P* > 0.05).

**Conclusion:**

PIVR combined with PKP can overcome the limitations of PKP alone, that is, hardly restoring vertebral height and height being easily lost again with balloon removal. The combined method can also restore the vertebral fractures to a satisfactory height and effectively maintain the stability of the spine, which improves the long-term quality of life of patients. Thus, PIVR combined with PKP is a better choice for patients with OVCFs.

## Background

With the aging of the population, the incidence of osteoporotic vertebral compression fractures (OVCFs) is getting higher and higher in clinics around the world, seriously threatening the life and health of elderly patients. OVCFs always cause chronic pain, depression, insomnia, and even loss of ability to perform daily activities [1]. At present, percutaneous kyphoplasty (PKP) is widely used for the treatment of OVCFs [2, 3]. PKP has the advantages of being minimally invasive and safe, providing rapid pain relief, and being a simple manipulation technique [4]. However, there are still some drawbacks, including unsatisfactory reduction, postoperative long-term loss of height, and kyphosis, especially for patients with severe OVCFs [5–7]. In order to achieve a better vertebral restorative effect, some scholars [8] have used open surgery with pedicle screws for distraction and fixation combined with vertebroplasty to treat OVCFs and have achieved satisfactory recovery of the collapsed vertebral body. However, the surgical trauma is substantial, and due to the poor bone quality of the patients, the holding force of the internal fixation is often insufficient, and the risk of postoperative failure is high. At the same time, the retention of the pedicle screw not only affects spinal activity but also may cause fractures of the adjacent vertebrae [9]. How to perfectly integrate the advantages of vertebral restoration and vertebroplasty is still a problem to be solved.

Therefore, we have developed a device to solve the above problems. This device has obtained a national invention patent. The current study was to compare the clinical effects of pedicle in vitro restorer (PIVR) combined with PKP with simple PKP surgery for the treatment of OVCFs. We hypothesized that the application of PIVR combined with PKP would perfectly restore the height of the vertebral body and correct kyphosis by using the method’s unique reduction technique.

## Materials and methods

### Clinical data

From August 2013 to August 2016, 72 patients with a single osteoporotic thoracic or lumbar vertebral compression fracture treated at Nanjing First Hospital, Nanjing Medical University, were selected. Seventy-two patients with OVCFs were treated surgically, with 30 patients undergoing PKP (group A) and 42 undergoing PIVR combined with PKP (group B). The procedures followed were in accordance with the ethical standards of Nanjing First Hospital’s committee, and consent was obtained from each patient (Permit Number KY20130201-02). All patients met the following inclusion criteria: (1) single vertebral fracture; (2) T10 and below vertebral fractures; (3) loss of vertebral body anterior column height of 30% or more, with no significant lesion to the middle and/or posterior columns; (4) age between 55 and 75 years old; (5) disease duration less than 3 weeks; and (6) bone mineral density value − 4.0 SD < *T* < − 2.5 SD. Patients with multi-segmental fractures, non-osteoporotic compression fractures, burst fractures, and fractures with spinal stenosis or spinal cord injury were excluded. In group A, there were 6 males and 24 females, and the ages ranged from 58 to 73 years, with an average age of 66.83 ± 4.90 years. In group B, there were 8 males and 34 females, and the ages ranged from 58 to 75 years, with an average age of 65.74 ± 4.65 years. The locations of the collapsed vertebrae were 58 lumbar vertebrae and 14 thoracic vertebrae, including 2 cases at T10, 4 cases at T11, 8 cases at T12, 22 cases at L1, 16 cases at L2, 13 cases at L3, and 7 cases at L4. In group A, the locations of the collapsed vertebrae were 5 thoracic vertebrae and 25 lumbar vertebrae. In group B, the locations of the collapsed vertebrae were 9 thoracic vertebrae and 33 lumbar vertebrae. The reasons for injuries were 16 cases of sprains, 12 cases of car accident injuries, 36 cases of tumbling injuries, and 8 cases of falling injuries. There were no significant differences in sex, age, and location of the collapsed vertebrae between the two groups (*P* > 0.05).

### Equipment and instruments

**Fig. 1:**
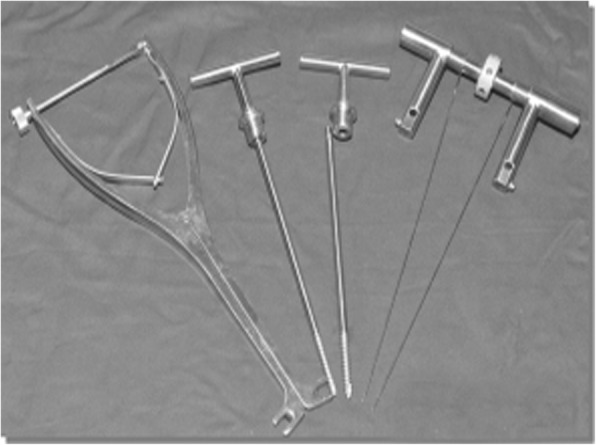
Picture of the PIVR

The minimally invasive equipment and the balloon were manufactured by the Kyphon Company of America, and the Flexiview 8800 C-type arm X-ray machine was manufactured by the GE Company of America. The PIVR was self-developed and has been awarded a national invention patent (Fig. 1 patent number: ZL200810123915.6).

### Surgical technique

**Fig. 2:**
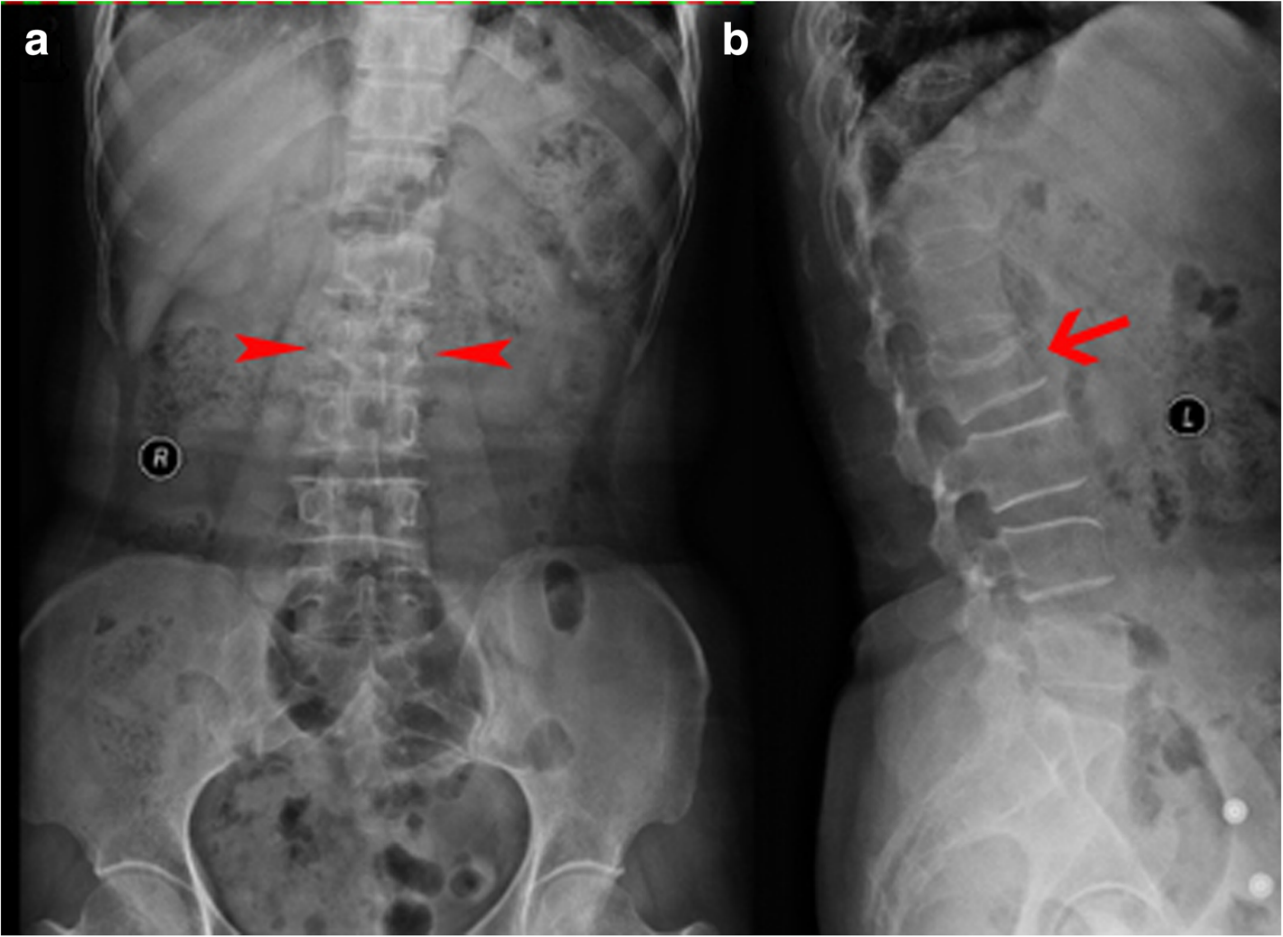
Typical radiographic views. **a**, **b** Preoperative anteroposterior and lateral radiographs showing a compression fracture of the second lumbar vertebra

For the group A procedure, patients were placed in the prone position with two pads for abdominal hanging, and the procedures were performed under local anesthesia with electrocardiogram monitoring; at the same time, fluoroscopy was used throughout the procedure. A small 8-mm incision was placed on the skin at the pedicle level, and the accurate incision position was on the outer edge of the pedicle’s projection under the anteroposterior view of the image. The needle was placed into the pedicle, and the needle pin was removed. Next, the guide pin was inserted into the first two thirds of the vertebral body in the lateral view; subsequently, a cannula was placed through the guide pin. The guide pin was pulled out, and the drill was inserted through the cannula to establish a surgical tunnel. The length of the tunnel in the vertebral body should be 3 mm larger than the length of the balloon after expansion. The balloon was inserted through the cannula and placed into the anterior three fourths of the vertebral body from a lateral view. The balloon was slowly inflated by injecting contrast media through the high-pressure pump. When the Cobb angle and vertebra’s height were satisfactory compared to preoperative radiographs (Fig. 2), the operator extracted the contrast media and withdrew the balloon. The same volume of bone cement, which became doughy, was injected into the collapsed vertebral body. When the bone cement was spread near the posterior wall of the vertebral body or appeared to leak, the procedure was stopped immediately. Contralateral puncture was performed simultaneously. Patients stayed in bed for 24 h.

**Fig. 3:**
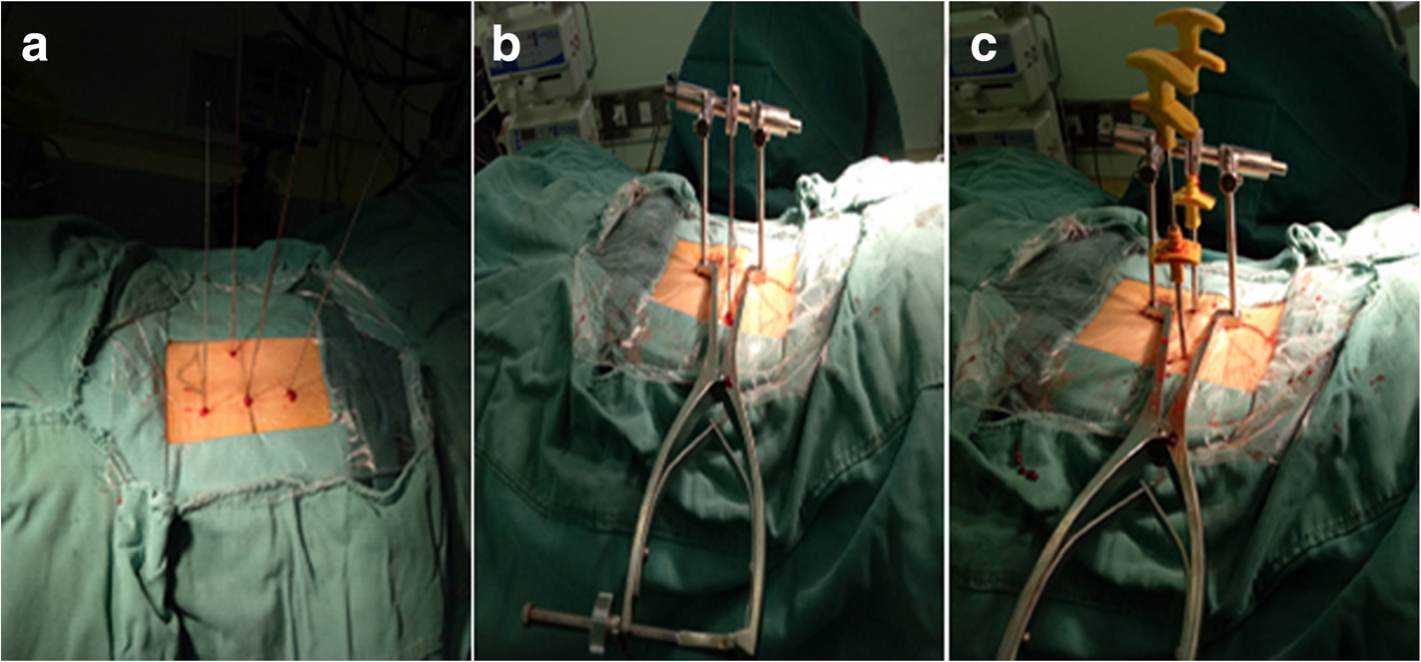
Three intraoperative photographs showing the operation process using PIVR. **a** Four pedicle screw guide pins are inserted into the pedicles via a minimally invasive percutaneous incision. **b** The restorer was used for distraction and restored the vertebral body. **c** PKP combined with PIVR

**Fig. 4:**
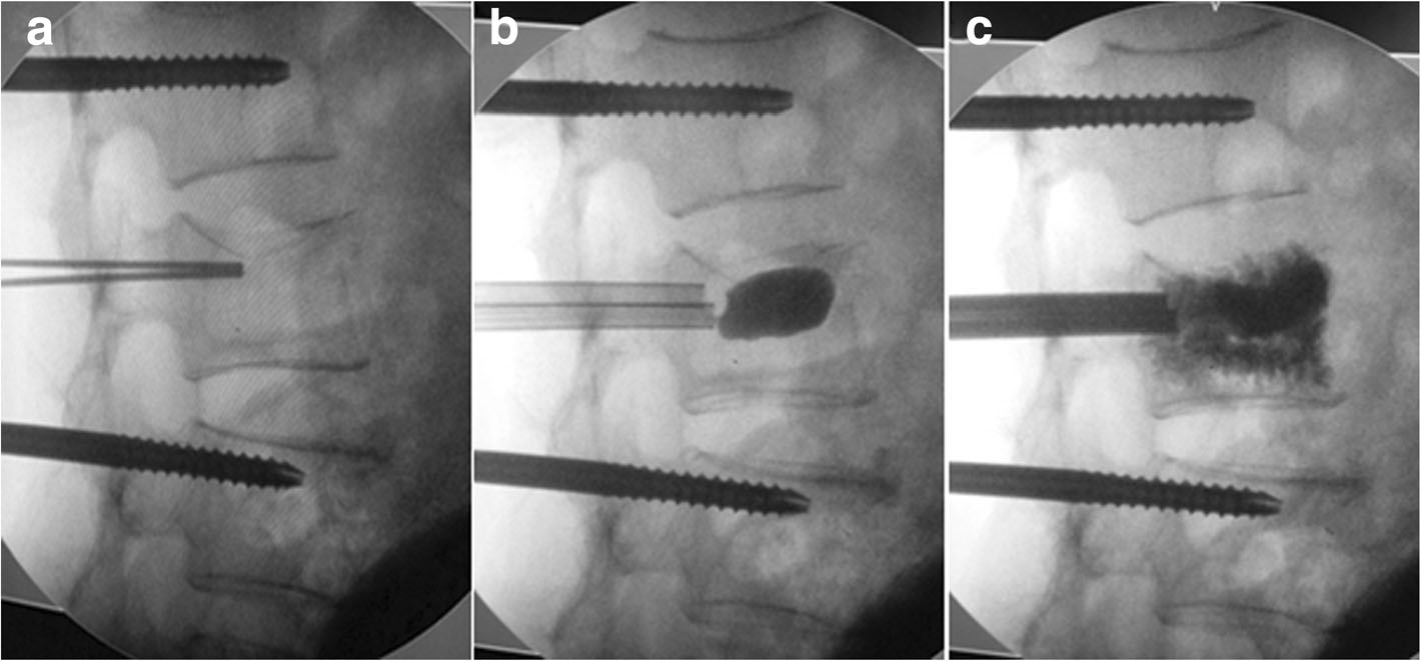
Intraoperative radiographs. **a** Distracting reduction using PIVR. **b** The balloon slowly inflates by injecting contrast media. **c** Bone cement being injected into the collapsed vertebral body

For the group B procedure, the patients were fixed in the prone position. PIVR was used to perform distraction on the side of the severe fracture of the vertebral body. If the collapse of the vertebral body was only in front and there was no significant difference in the bilateral height, the operation was performed on the left or right side according to the operator’s habits. Pedicle needles were implanted into the adjacent vertebral bodies around the collapsed vertebral body through minimally invasive percutaneous implantation under fluoroscopy (Fig. 3a). Subsequently, hollow pedicle screws were inserted one third of the way into the vertebral body along the positioning needle, and the distraction device was placed below the extension rod. Next, the distraction device was used to restore the vertebra (Figs. 3b and 4a); at the same time, the hex rotator of the positive and reverse threads was tightened to prevent the restorer from retreating. Under fluoroscopy, when the vertebral body reset was satisfactory or a screw-cutting phenomenon occurred, the reset procedure was terminated, and PKP was performed (Figs. 3c and 4b, c) with the application of a bilateral puncture technique; then, the restorer was removed. Patients stayed in bed for 24 h.

### Clinical and radiographic assessment

The improvement of anterior and mid-vertebral heights and the sagittal Cobb angle, which was defined as the crossing angle of the vertical lines parallel to the collapsed vertebral superior and inferior endplates in the lateral X-ray image, were the observed indices of the effect of restoration. A visual analogue scale (VAS) [10] was used to assess back pain control, and the Oswestry Disability Index (ODI) [11] was used to estimate the activities of daily living. Sexual life was deleted; as a result, the remaining 9 items totaled 45 points, according to living habits and age. Considering that there may be a mechanical imbalance in the unilateral distraction of the restorer, the lateral wall height improvement ratio on the distraction side of the restorer was compared with that of the non-distraction side. Lateral wall height improvement ratio = (postoperative height − preoperative height)/postoperative height × 100%.

### Statistical analysis

Statistical analyses were performed using SPSS version 19.0 statistical software (IBM SPSS, Chicago). Quantitative data are displayed in the form of $\overline{x}\pm s$. The values between groups were analyzed by independent sample *T* test. Preoperative and postoperative values between different subgroups were compared using the paired *T* test. Enumeration data between groups were analyzed by chi-square test. The results were considered significant at *P* < 0.05.

## Results

Seventy-two vertebrae were successfully injected with bone cement, with negligible blood loss, no deaths, no spinal cord injuries, and no pulmonary embolisms or postoperative infections. Sixty-three patients were followed up. Two patients were followed up for 3 months and actively withdrew from follow-up. Five patients actively withdrew from follow-up at 6 months. Two patients withdrew from follow-up due to relocation. Eight asymptomatic extravasations of vertebral bone cement occurred, including 2 cases of intervertebral space, 2 cases of paravertebral vein, and 3 cases of paravertebral soft tissue in group A and 1 case of paravertebral vein in group B. There were significant differences in the ratio of cement leakage between the two groups (*P* < 0.05). The anterior and mid-vertebral body heights for group A increased from 18.23 ± 1.11 mm and 19.80 ± 1.05 mm preoperatively to 22.61 ± 1.21 mm and 23.88 ± 1.05 mm 1 week postoperatively, respectively. The anterior and mid-vertebral body heights for group B increased from 17.67 ± 1.18 mm and 19.17 ± 1.04 mm preoperatively to 23.32 ± 1.14 mm and 24.34 ± 0.97 mm 1 week postoperatively, respectively. Cobb angles between group A and group B improved from 20.87° ± 1.32° and 22.60° ± 1.43° before surgery to 10.84° ± 1.03° and 10.51° ± 0.77 1 week after surgery, respectively; VAS between the two groups decreased from 8.03 ± 0.40 and 8.01 ± 0.37 before surgery to 3.01 ± 0.35 and 2.35 ± 0.28 1 week after surgery, respectively. ODI at the last follow-up in group A (17.69% ± 4.60%) was significantly different than that in group B (11.71% ± 2.20%) (*P* < 0.05). There was no significant difference in the incidence of recurrent vertebral fractures between the two groups at last follow-up (*P* > 0.05) (Tables 1 and 2). There was no significant difference in the improvement ratio of the height of the lateral wall of the vertebral body between the distraction side and non-distraction side of the restorer (*P* < 0.05) (Table 3).

**Table 1:** Preoperative and postoperative characteristics of the two groups

Characteristic	Group A	Group B	*t*/*χ*^*2*^	*P*
No. (male:female)	30 (6:24)	42 (8:34)	0.010	> 0.05
Mean age, year	66.83 ± 4.90	65.74 ± 4.65	0.964	> 0.05
Mean improvement of anterior height, mm	4.38 ± 0.59	5.66 ± 0.64	− 8.549	< 0.05
Mean improvement of mid height, mm	4.09 ± 0.27	5.17 ± 0.49	− 11.938	< 0.05
Mean improvement of VAS	5.01 ± 0.22	5.66 ± 0.29	− 10.353	< 0.05
Mean improvement of Cobb angle, deg	10.03 ± 0.52	11.08 ± 1.12	− 10.380	< 0.05
The ratio of bone cement leakage, %	23.33	2.38	5.802	< 0.05

*No.* number, *deg* degrees, *VAS* visual analogue scale

**Table 2:** Characteristics of the two groups at last follow-up

Characteristic	Group A	Group B	*t*/*χ*^*2*^	*P*
Mean ODI, %	17.69 ± 4.60	11.71 ± 2.20	6.157	< 0.05
No. (refracture:normal)	26(2:24)	37(1:36)	0.099	> 0.05

*No.* number, *ODI* Oswestry Disability Index

**Table 3:** Comparison of the lateral wall height improvement ratio by the restorer

Group B	*n*	Lateral wall height improvement ratio, %	*t*	*P*
Distraction side	42	22.75 ± 2.10	1.18	0.24
Non-distraction side	42	22.20 ± 2.22		

## Discussion

PKP has opened up a new way for the treatment of OVCFs in the elderly. However, the dilator expands in the collapsed vertebral body with greater resistance, and there is limited space for expansion. In addition, the height of vertebral body cannot be completely maintained under the compression of the adjacent upper and lower vertebral bodies after the balloon is withdrawn from the vertebral body, and the height of the vertebral body is easily lost again. PKP may also be associated with paraspinal muscle tension and traction because of local anesthesia [12]. The above factors have an influence on the height of collapsed vertebrae, which cannot be fully restored. So, kyphosis of the spine can only be partially rectified. Therefore, we developed a set of special equipment, named PIVR.

The principle of PIVR is a hollow elongated pedicle screw and its self-locking compression-distraction device, with large arms. The pedicle screw is 15 cm long, with a hollow diameter of 1.2 mm and outer diameter of 6.5 mm, 6.0 mm, 5.5 mm, or 5.0 mm. The self-locking compression-distraction device includes a screw-tail prolonged rod and a distraction prolonged rod. The hollow structure of the pedicle screw allows easy implantation of the minimally invasive percutaneous needle and hollow taps; the hollow screw is longer than common screws used for internal fixation. Moreover, the rod at the screw tail prolongs the length of the extension arm. The hollow pedicle screw is located at the adjacent vertebrae and can exert its powerful distraction and compression effect through its long arm, resetting the collapsed vertebrae according to the principle of ligament reconstruction [13]. The distraction and compression effects are mediated via distraction and compression devices, which are equipped with positive and reverse screw threads (thread bar); the thread bar is vertically connected to the prolonged rod. The hex rotator is fixed on the thread bar, and rotation of the hex rotator can shorten the distance between the two prolonged rods, thus functioning for distraction and reduction.

Reconstruction of the vertebral body by balloon during PKP surgery has a very limited effect on restoration, which may be related to the following factors: (1) the direction of balloon expansion is difficult to control, (2) the volume and expansion capacity of the balloon itself is limited (the working tension of the balloon is generally less than 300 psi), and (3) after the balloon is removed from the vertebral body during the operation, fracture reduction is difficult to maintain. However, PIVR, through the self-locking distraction device to maintain the distraction state, can overcome easy loss of vertebral body height after withdrawal of the balloon and can effectively restore the height and correct the kyphosis. There were significant differences between the two groups in the improvement of the height of the vertebral body and Cobb angle 1 week postoperatively (*P* < 0.05), and the improvements in group B were better than those in group A. Some patients undergoing PKP surgery suffer from “stress concentration” due to poor reduction of vertebral fractures caused by collapsed fractures and adjacent vertebrae, which may lead to long-term complications of low back pain and affect quality of life. However, PIVR can effectively maintain the stability of the spine, which improves the long-term quality of life of patients. ODI at the last follow-up in group A (17.69% ± 4.60%) was significantly different than that in group B (11.71% ± 2.20%) (*P* < 0.05). It showed that the long-term clinical effect for group B was significantly better than that for group A.

PVP and PKP can effectively reduce pain. He et al. [3] reported the follow-up results of an average of 58 months (24.1–98.9) in 11 cases of PVP, and all patients were significantly relieved of pain, with significant long-term pain relief and no restriction of daily activities. In our study, the pain in each group was alleviated to different degrees, and the improvement of VAS in group A postoperatively was less than that in group B. PIVR had a good reduction effect on the collapsed vertebrae; as a result, bone cement fully infiltrated. Additionally, bone cement filling can effectively block blood supply and lead to nerve ending necrosis, which results in a more satisfactory analgesic effect [14].

Bone cement leakage and adjacent vertebral fractures are the common complications of PKP. Zhan et al. [15] concluded that the leakage rate of bone cement in PVP and PKP was 54.7% and 18.4%, respectively, through 22 meta-analyses of 2872 cases. Lieberman [16] reported 70 cases of PKP in 30 patients where the incidence of bone cement leakage was 8.6%. Xie et al. believed that the leakage of bone cement may be related to patient age, bone density, vertebral cortical defect, bone cement viscosity, bone cement injection speed, and time [17]. In our study, the bone cement leakage rate in group A (23.33%) was higher than that in group B (2.38%). There was a significant difference in the ratio of bone cement leakage between the two groups (*P* < 0.05). PIVR has a powerful distraction effect on collapsed vertebra. It promotes the reduction of the compressed height and increases the volume of the compressed bone tissue, restoring bone density of the compression zone to normal. The increase in bone tissue volume reduces the pressure during the injection of bone cement, which leads to a decrease in the bone cement leakage rate. Although the recovery of vertebral body stiffness is closely related to the amount of bone cement, it is controversial whether more bone cement injections will result in better treatment outcomes [18, 19]. Some scholars believe that excessive bone cement injection can cause fractures in adjacent vertebrae. Similarly, different scholars hold different perspectives on the factors that cause fractures in adjacent vertebral bodies [20–23]. Yang et al. [24] showed that the injection of a large amount of bone cement, bone cement leakage, and severe osteoporosis in patients were risk factors for adjacent vertebral fracture. In our study, there were no significant differences in the incidence of recurrent vertebral fractures between the two groups at the last follow-up (*P* > 0.05).

Considering that there may be a mechanical imbalance in unilateral distraction, this study specifically compared the improvement ratio of the height of the lateral wall of the vertebral body on the side of the restorer as compared with that of the non-distraction side, and the results showed that there was no statistically significant difference between the two sides (Table 3). There are three possible reasons. (1) Intraoperative adjustment was based on the reduction of the restorer. If the height of the non-distraction side was found to be poorly reset, the balloon should be inflated as much as possible to reduce the angulation of the vertebral body. In addition, if lateral compression of the vertebral body was found to be uneven intraoperatively, the severe side wall was selected to distract. (2) OVCFs were mostly low-energy injuries, even if they were unilaterally stretched, and the fractures were easily reset. (3) In the case that the middle and posterior columns were relatively intact, under the action of the tensile stress of the annulus fibrosus and the anterior and posterior longitudinal ligaments, the collapsed vertebral body was more evenly balanced; generally, no lateral angulation occurred.

PIVR combined with PKP should avoid the cutting effect of screws because of excessive distraction, which causes iatrogenic fractures. There were various reasons for the absence of iatrogenic vertebral fractures in group B. First, the process of resetting was under X-ray to observe whether there was a light zone between the screws and the vertebral body; the reset should be stopped immediately once a light zone is observed. Second, patients with severe osteoporosis (*T* < − 4.0 SD) were not included in the study. Lastly, the sample size of this study was small, and more studies need to be conducted with PIVR to observe its complications.

There are some limitations in the application of PIVR combined with PKP. Only thoracic and lumbar segmental vertebral fractures can be treated because thoracic protection of the vertebrae above T10 is not required for reduction. The course of illness after injury is less than 3 weeks; otherwise, it is difficult to recover from surgery. The operation requires another two punctures in the adjacent vertebral body, which increases new trauma, but postoperative VAS did not show an increase in pain. For the case of multiple vertebral fractures, PIVR is difficult to operate, and multiple vertebral fractures require multiple operations. For patients with severe osteoporosis, the nail rod is easily made unstable, and the holding force of pedicle screws is insufficient; as a result, the effect of distraction is poor. Additionally, it is easy to form iatrogenic fractures due to the screw-cutting effects of severe osteoporosis.

## Conclusion

PIVR has great fatigue performance regarding the biomechanics in PKP and can restore physiological curvature and mechanical strength of the spine effectively, overcoming the weaknesses of PKP restoring the vertebral height and easily losing the height of the vertebral body after withdrawal of the balloon. Especially for patients with significant compression of the vertebral body (more than 1/3), the effect of the surgery is obvious. PIVR can restore the vertebral fractures to a satisfactory height, effectively maintaining the stability of the spine and significantly improving the quality of life of patients. PIVR combined with PKP is a better treatment option.

## Abbreviations

ODI: Oswestry Disability Index
OVCFs: Osteoporotic vertebral compression fractures
PIVR: Pedicle in vitro restorer
PKP: Percutaneous kyphoplasty
VAS: Visual analogue scale
